# Case Report: Therapeutic effect of hypofractionated radiotherapy using HyperArc for giant cavernous sinus hemangiomas

**DOI:** 10.3389/fonc.2025.1557291

**Published:** 2025-10-16

**Authors:** Mengqi Yang, Zhaoming Peng, Xin Li, Baodong Chen, Feng Peng, Yajie Liu

**Affiliations:** ^1^ Department of Radiation Oncology, Peking University Shenzhen Hospital, Shenzhen, China; ^2^ Department of Neurosurgery, Peking University Shenzhen Hospital, Shenzhen, China

**Keywords:** hemangioma, hypofractionated radiotherapy, HyperArc, complications, disease control

## Abstract

**Background:**

Cavernous sinus hemangiomas (CSH) are considered benign vascular skull base tumors. Surgical therapy is the primary treatment due to the neurological deficits resulting from the compressive effects of the mass. However, patients with tumors located in critical areas or those with large tumor volume, surgical resection may be difficult or even unfeasible.

**Case Description:**

We report the case of a female patient with a 10-year history of progressive right orbital swelling and visual impairment. Imaging revealed a giant CSH with a tumor volume of 144 cm³. The patient underwent limited debulking surgery one year prior, with substantial residual tumor burden and persistent right facial swelling, particularly in the periorbital region.

**Intervention:**

The patient subsequently received hyperarc-based hypofractionated radiotherapy, delivered at a dose of 30 Gy in 10 fractions (3 Gy per fraction), followed by clinicoradiologic assessments at 6-month intervals.

**Outcome:**

Radiologic evaluation at 6 months post-radiotherapy demonstrated a 69% reduction in tumor volume. Significant improvement in facial swelling and restoration of facial symmetry were observed. However, right-sided ptosis and vision loss persisted, likely due to irreversible optic nerve damage. A transient alopecia noted at 2 months post-treatment resolved completely by the 6-month follow-up. No acute or late radiation-related toxicities were reported during a 13-month follow-up period.

**Conclusion:**

The treatment achieved marked tumor regression and clinical improvement with an excellent safety profile. Hypofractionated radiotherapy may be serve as an alternative effective approach in these unresectable lesions with a favorable safety profile.

## Introduction

Cavernous sinus hemangiomas (CSH) are well-defined neoplasms that originate within the dural inuses, typically located in the intracranial extradural space. The orbital apex and superior orbital fissure are commonly affected sites. Due to the complex anatomy of this region and the associated risks, surgical management of CSH remains highly challenging. Although surgery is still considered the gold standard, radiosurgery has emerged as a valid option for treating residual tumors (1). Several studies have demonstrated favorable responses of small and medium-sized CSH to gamma knife radiosurgery (GKRS) (2). For giant CSH (>4 cm), GKRS may also serve as an effective alternative treatment modality (3). There are no reports in the global literature documenting the use of primary hypofractionated radiotherapy for giant CSH.

HyperArc (HA), delivered via the Varian TrueBeam linear accelerator, is a novel non-coplanar multi-arc radiotherapy technique that has shown considerable promise in the treatment of head and neck tumors as well as intracranial metastases (4). The HyperArc planning system enables safe dose escalation while achieving optimal target coverage. Several studies have highlighted its ability to significantly improve target dose uniformity, generate sharp dose gradients outside the target, and minimize radiation exposure to organs at risk (OARs), including the lens, retina, and optic nerve (5–7). These advantages contribute to a decreased incidence of radiation-induced adverse effects such as vision loss, blindness, and corneal ulceration during radiotherapy (RT).

Here, we present a case of a giant CSH treated with hypofractionated radiotherapy using HA technology. In our case, extensive surgical resection posed a substantial risk of damaging peripheral organs such as the eyes and appendages. Therefore, the patient underwent hypofractionated HA-based radiotherapy following palliative debulking surgery. Despite the tumor remained radiographically visible after treatment, the radiotherapy successfully arrested tumor growth, restored facial symmetry, and improved the patient’s quality of life.

## Case report

In 2013, a 20-year-old female with no significant past medical history presented with progressive swelling of the right eye and gradual visual decline. The patient denied ocular pain, facial numbness, or other neurological deficits. The first medical consultation and imaging evaluation took place in 2015. The initial magnetic resonance imaging (MRI) (**Supplementary Figure 1**) revealed circumscribed retro-orbital mass located posterior to the right globe. The mass compressed the right eyeball anteriorly and encased the optic nerve, leading to disruption of both the optic nerve and surrounding extraocular muscles. On T1-weighted images (T1WI), the lesion appeared isointense relative to adjacent muscles, while T2-weighted images (T2WI) demonstrated heterogeneous hyperintensity with clustered components. Mild, heterogeneous contrast enhancement was observed. The mass extended into the cavernous sinus, posterior longitudinal fissure, right cerebellar tentorium, and subcutaneous temporal soft tissues, raising a preliminary radiologic impression of a giant cavernous sinus hemangioma (CSH). The patient had not received any treatment at that time. The patient experienced progressive worsening of proptosis and right-sided facial swelling. A repeat MRI in 2019 confirmed significant enlargement of the lesion with persistence of the same anatomical involvement (**Supplementary Figure 2**).

**Figure 1:**
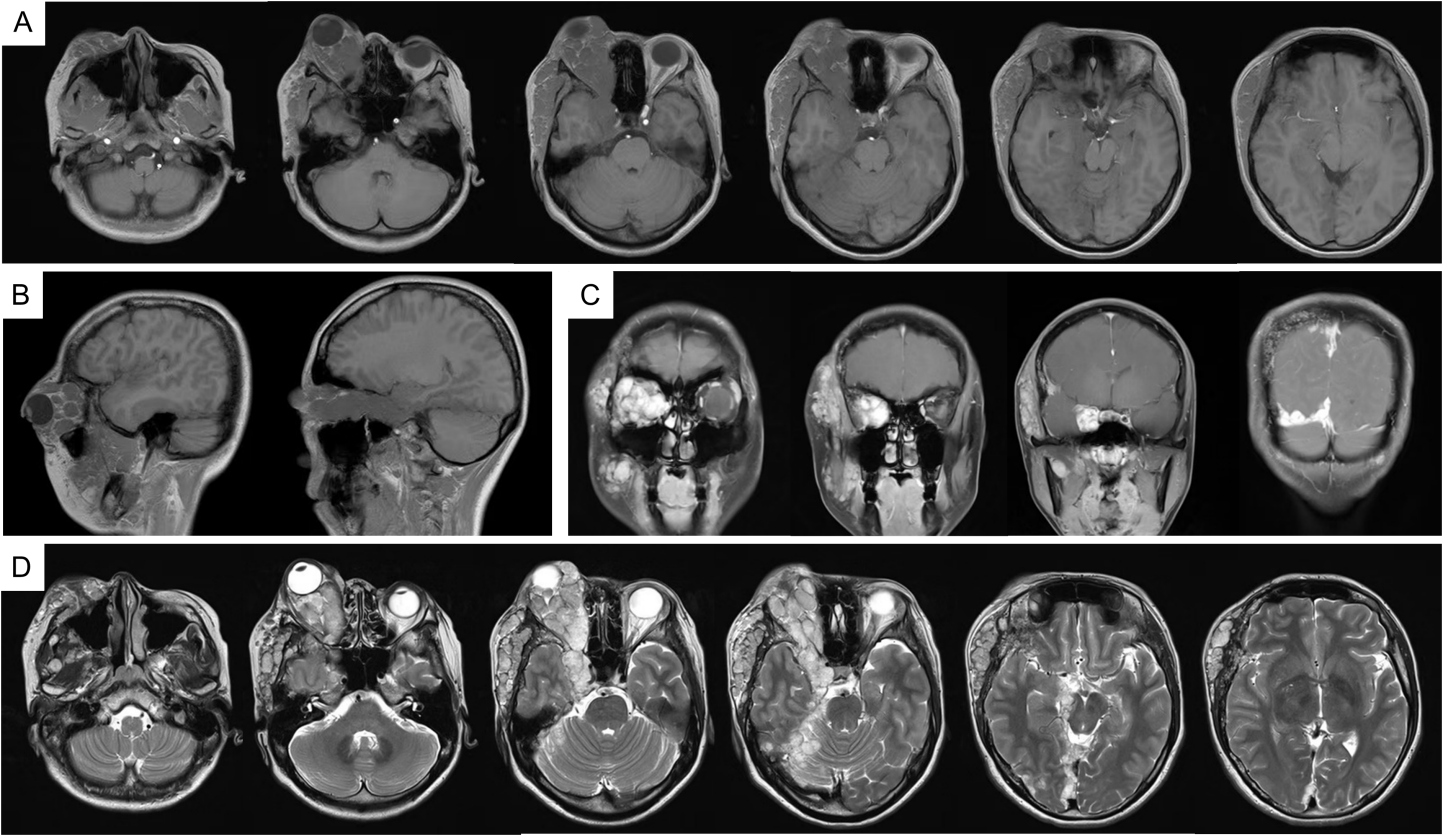
MRI imaging in 2023. **(A)** T1WI; **(B)** T1WI-C coronal view; **(C)** T1WI-C sagittal view; **(D)** T2WI-TSE. MRI, magnetic resonance imaging; T1WI, T1-weighted image; T2WI, T2-weighted image; T1WI-C, T1WI with contrast; T2-FLAIR, T2-fluid attenuated inversion recovery.

**Figure 2:**
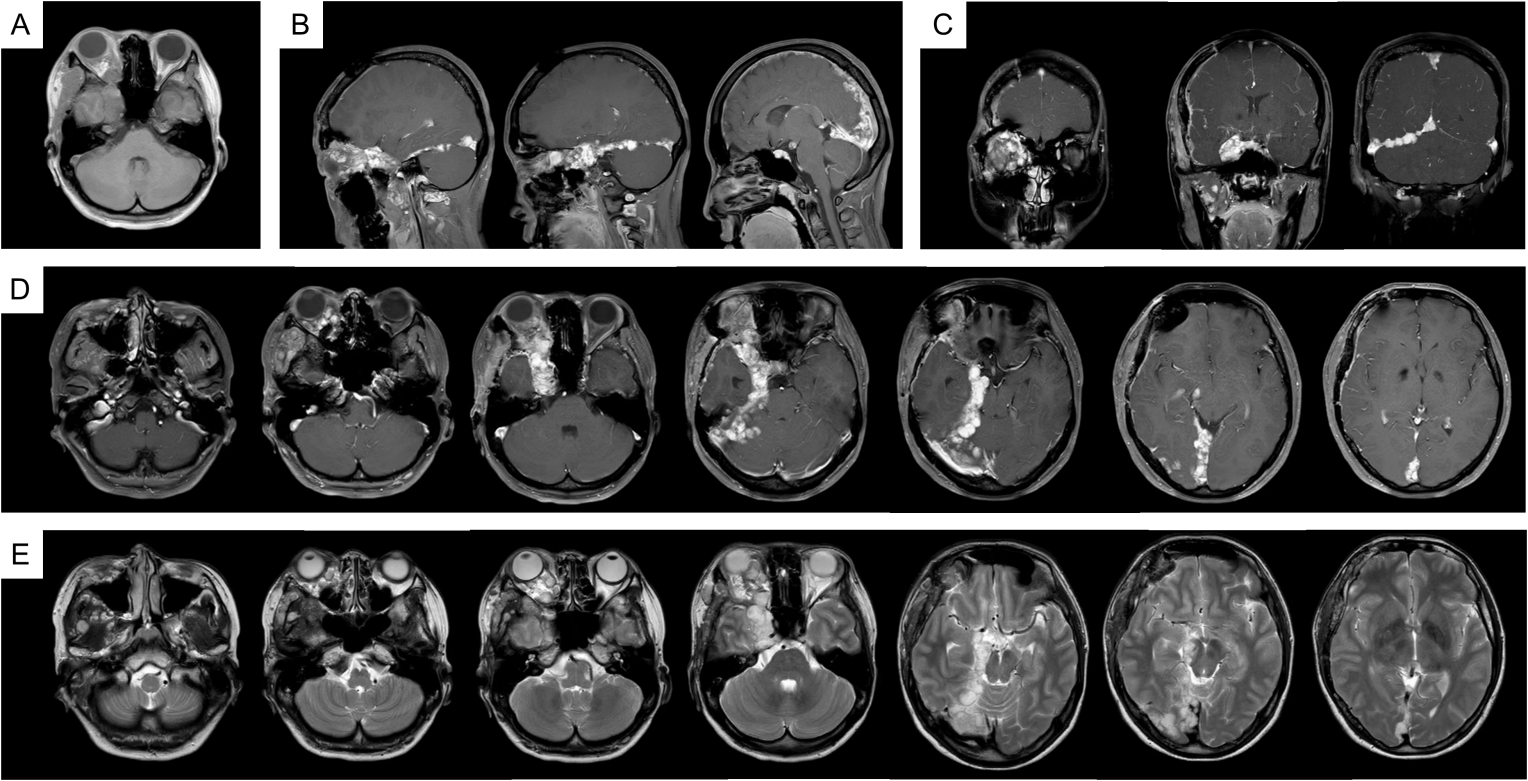
MRI imaging after the palliative surgery. **(A)** T1WI; **(B)** T1WI-C coronal view; **(C)** T1WI-C sagittal view; **(D)** T1WI-C; **(E)** T2WI-TSE.

In 2023, due to worsening symptoms and brainstem compression (**Figure 1**), palliative surgical resection was performed. Multiple tumor nodules beneath the temporal muscle and intraorbital lesions were partially removed. Postoperative histopathological analysis confirmed the diagnosis of hemangioma, characterized by dilated vascular channels lined by a single layer of endothelial cells without atypia, consistent with a cavernous subtype. This pathological confirmation established the final diagnosis, thereby excluding other radiologically plausible differential diagnoses such as meningioma (which typically shows dural attachment and uniform enhancement), schwannoma (which often arises from cranial nerves with distinct borders), or arteriovenous malformation/hemangioblastoma (usually associated with flow voids or feeding vessels). At four months post-surgery, follow-up MRI (**Figure 2**) showed partial improvement of right cerebral edema, though a substantial intracranial tumor burden persisted.

**Figure 3:**
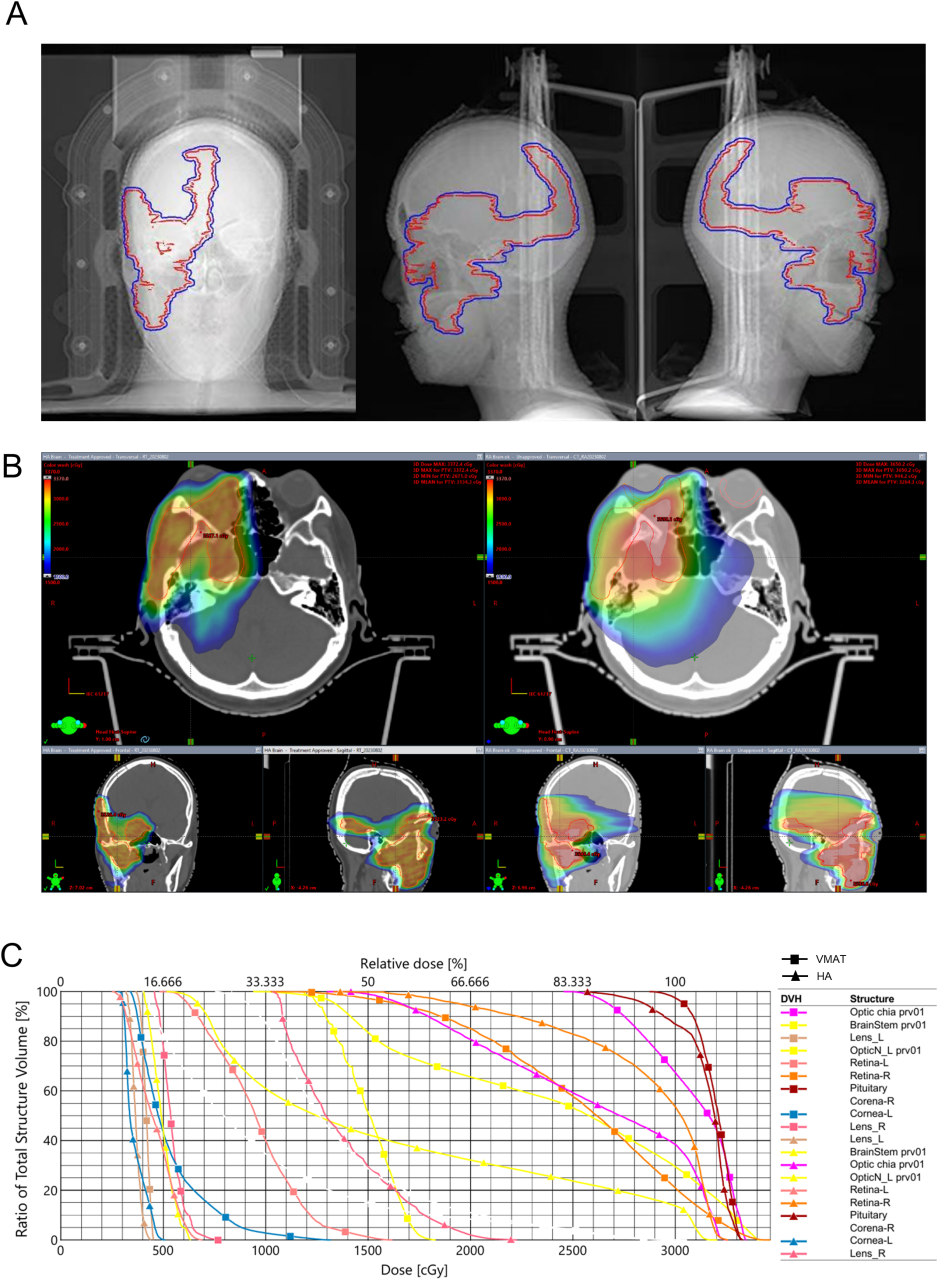
Treatment plan and spatial dose distribution comparison. **(A)** red: GTV, blue: PTV; **(B)** HA; **(C)** VMAT; **(D)** DVH. GTV, gross tumor volume; PTV, planning target volume; HA, HyperArc; VMAT, volumetric-modulated arc therapy; DVH, dose volume histogram.

Given the residual disease and progressive natural history, postoperative radiotherapy was recommended. Hypofractionated radiotherapy using HA technology was selected to minimize radiation dose to organs at risk (OARs), including the cornea, optic nerves, and other critical structures within the cavernous sinus (**Table 1**). Compared with volumetric modulated arc therapy (VMAT), the HA treatment plan demonstrated superior conformity, steeper dose fall-off, and improved homogeneity, with favorable indices including conformity index (CI), gradient index (GI), and homogeneity index (HI) (**Table 2**). Gross tumor volume (GTV) and planning target volume (PTV) were shown in **Figure 3A**. The spatial dose distribution differences between HA and VMAT are illustrated in **Figures 3B, C**.

**Table 1:** The maximum and mean dose (cGy) of OARs.

RT	Brainstem Dmax	Len_L Dmax	Cornea_L Dmax	Retina_L Dmax	Pituitary Dmax	Optic nerve_L Dmax	Parotid_L Dmean
HA VMAT	3204 3420	440 452	511 1319	691 1619	3327 3328	634 1830	297 692

OAR, organ at risk; RT, radiotherapy; Dmax, maximum dose; Dmean, mean dose; VMAT, volumetric-modulated arc therapy; HA, HyperArc.

**Table 2:** The calculations of radiation dosimetry (the calculation formulas of dosimetric parameters are shown in **Supplementary Materials** and **methods**).

RT	RTOG CI	Paddick CI	GI	HI
HA VMAT	1.12 1.22	0.85 0.51	2.58 3.38	0.08 0.25

RTOG, Radiation Therapy Oncology Group; CI, conformity index; GI, gradient index; HI, homogeneity index; VMAT, volumetric-modulated arc therapy; HA, HyperArc.

**Figure 4:**
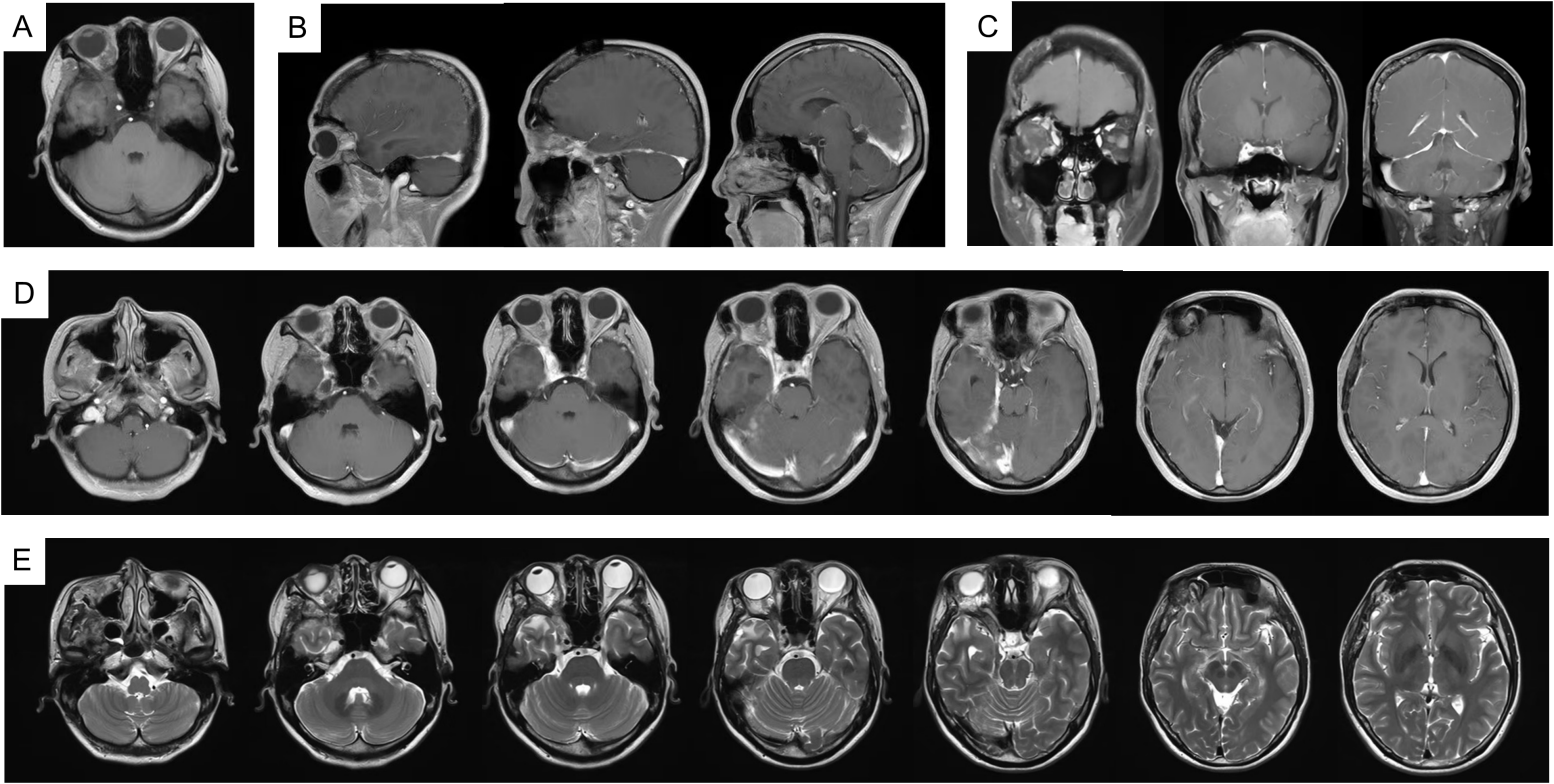
MRI imaging at 13 months follow up radiology. **(A)** T1WI; **(B)** T1WI-C coronal view; **(C)** T1WI-C sagittal view; **(D)** T1WI-C; **(E)** T2WI-TSE.

**Figure 5:**
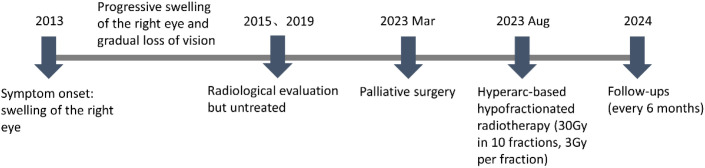
The clinical timeline of this patient.

In fact, the patient tolerated the radiotherapy well and experienced no significant adverse effects during treatment. At two months post-radiotherapy, marked improvement in right facial swelling was observed. MRI confirmed a reduction in both subcutaneous soft tissue swelling and cerebral hemisphere edema (**Supplementary Figure 3**). Follow-up evaluations were conducted every six months. At six months post-radiotherapy, imaging showed no evidence of disease progression (**Supplementary Figure 4**). Encouragingly, there was 69% and 79% volume reduction at six and thirteen months. The patient’s facial symmetry has been restored and there has been no evidence of tumor progression (**Figure 4**), signifying the effectiveness and tolerability of the treatment. Unfortunately, the patient has persistent ptosis of the right eyelid and unrestored vision due to irreparable neurological damage. Postoperative hypofractionated HA radiotherapy was administered for the patient to address the remaining tumor and prevent further progression. The clinical timeline of this patient was illustrated in **Figure 5**.

## Discussion

The primary treatment approach for large and giant CSH is surgical intervention, including radical or extensive resection. However, the rate of complete resection remains low, and postoperative deterioration in cranial nerve function is relatively common (8–10). Adjuvant radiotherapy is often administered following surgery to maximize the eradication of residual lesions. The optimal treatment strategy for large and giant CSH remains under debate. There has been a growing trend to consider radiosurgery as the primary treatment modality for CSH, particularly in the case of small to medium-sized lesions. Several studies have demonstrated favorable outcomes in terms of tumor shrinkage and sustained long-term control in CSH patients treated with GKRS (11–13). One study reported that large-volume CSHs could be safely treated with hypofractionated GKRS, achieving remarkable volumetric reductions over a short period (3). This suggested that radiosurgery alone may be a viable alternative to surgical intervention. In summary, radiotherapy remains a crucial modality for achieving local control and preventing recurrence in the management of CSH; however, its efficacy for giant lesions remains uncertain, posing a challenge in defining optimal treatment regimens.

In head and neck radiotherapy, VMAT is commonly employed as the standard technique (14). In the present case, the tumor’s invasion into the orbit, cavernous sinus and intracranial required a treatment plan with a high degree of target conformity and a steep dose gradient. The tumor’s proximity to vital structures, such as the eyeball, posed significant challenges in balancing effective tumor coverage with sparing of OARs. As showed in **Figure 5**, the comparison of radiotherapy plans for HA and VMAT exhibited significant differences in spatial dose distributions. The isodose lines for the HA plan exhibited superior closure compared to VMAT. The calculations suggested that HA outperformed VMAT in terms of CI, GI and HI. Notably, HA resulted in lower radiation doses to critical OARs, providing enhanced protection for structures such as the cornea, retina, optic nerve, parotid and brainstem. This reduction in radiation exposure is crucial in minimizing the risk of treatment-related complications. Published studies have also demonstrated HA plans generated a lower dose to the OARs and the normal tissue (15, 16). In our opinion, HA played a pivotal role in reducing adverse reactions during the radiotherapy course.

Indeed, the patient experienced no notable side effects during the entire course of radiotherapy. Unfortunately, her vision could not be restored after treatment. The persistent ptosis of the right eyelid and visual loss are likely due to irreversible damage to the optic apparatus and cranial nerves III to VI caused by the tumor itself. At the 13-month follow-up, the tumor volume had reduced by 69%. The patient exhibited no new neurological deficits, and no radiation-induced side effects were observed on MRI. The lesions of our patient were located in the retro-orbital space, right masseter muscle, cavernous sinus region, posterior longitudinal fissure of the brain, and the right cerebellar tentorium. GKRS or higher prescription dose for such large region would pose a risk for OARs and further cause any severe complications. The hypofractionated radiotherapy was considered as a treatment option in view of the giant size of the lesion.

A single dose of 3 Gy was unlikely to cause significant damage to surrounding OARs. The total dose of 30 Gy remained below the tolerance thresholds for most intracranial organs. Importantly, the relatively low cumulative dose allows for the possibility of future radiotherapy in the event of tumor recurrence. Due to the size and extent of the lesions, complete eradication through surgery or radiotherapy alone remains challenging. Thus, there is a high risk of future recurrence. Treatment planning should not only address the current disease status but also anticipate long-term management. It is also important to consider potential therapeutic options should the disease progress. In the future, genetic profiling of oncogenic and other relevant genes may help elucidate the molecular mechanisms underlying CSH pathogenesis. This evaluation could also inform potential gene therapy applications, including the endovascular *in situ* delivery of chemotherapeutics (17). Regardless, given the limited previous reports on treatment strategies for this giant condition, our approach provides valuable clinical insight. The success observed in this case contributes meaningful data and may guide clinicians facing similar therapeutic challenges.

## Limitations

Although our study highlights the safety and efficacy of HA−based hypofractionated radiotherapy for intracranial lesions, certain limitations remain. The most significant limitation is that the report is based on a single case of giant CSH with a follow-up duration of only 13 months. Longer-term follow-up is essential to assess the durability and full efficacy of this treatment approach. Additionally, findings from a single case cannot provide definitive evidence that HA achieves superior local control or survival benefits. Larger studies with extended follow-up are necessary to validate these preliminary observations.

## Conclusion

Giant CSH presents a significant challenge, particularly when intracranial structures are involved. Given the absence of an established optimal treatment strategy, there remains an urgent need for more effective therapeutic options. In this case, a combination of palliative debulking surgery followed by hypofractionated HA radiotherapy was employed. Our findings suggest that this strategy was effective in achieving tumor control and improving patient outcomes. Furthermore, HA demonstrated superior target coverage and better sparing of critical structures, indicating its potential clinical advantage. However, further studies are needed to confirm these findings in a larger patient cohort.

## Data Availability

The original contributions presented in the study are included in the article/**Supplementary Material**. Further inquiries can be directed to the corresponding authors.
