# Supplementary material for: Case Report: Therapeutic effect of hypofractionated radiotherapy using HyperArc for giant cavernous sinus hemangiomas
Source: Front Oncol. 2025 Oct 16;15:1557291. doi: 10.3389/fonc.2025.1557291 (PMC12571567; doi:10.3389/fonc.2025.1557291)
Supplement: Supplementary file 1 [file DataSheet1.docx]

**Supplementary Figures**


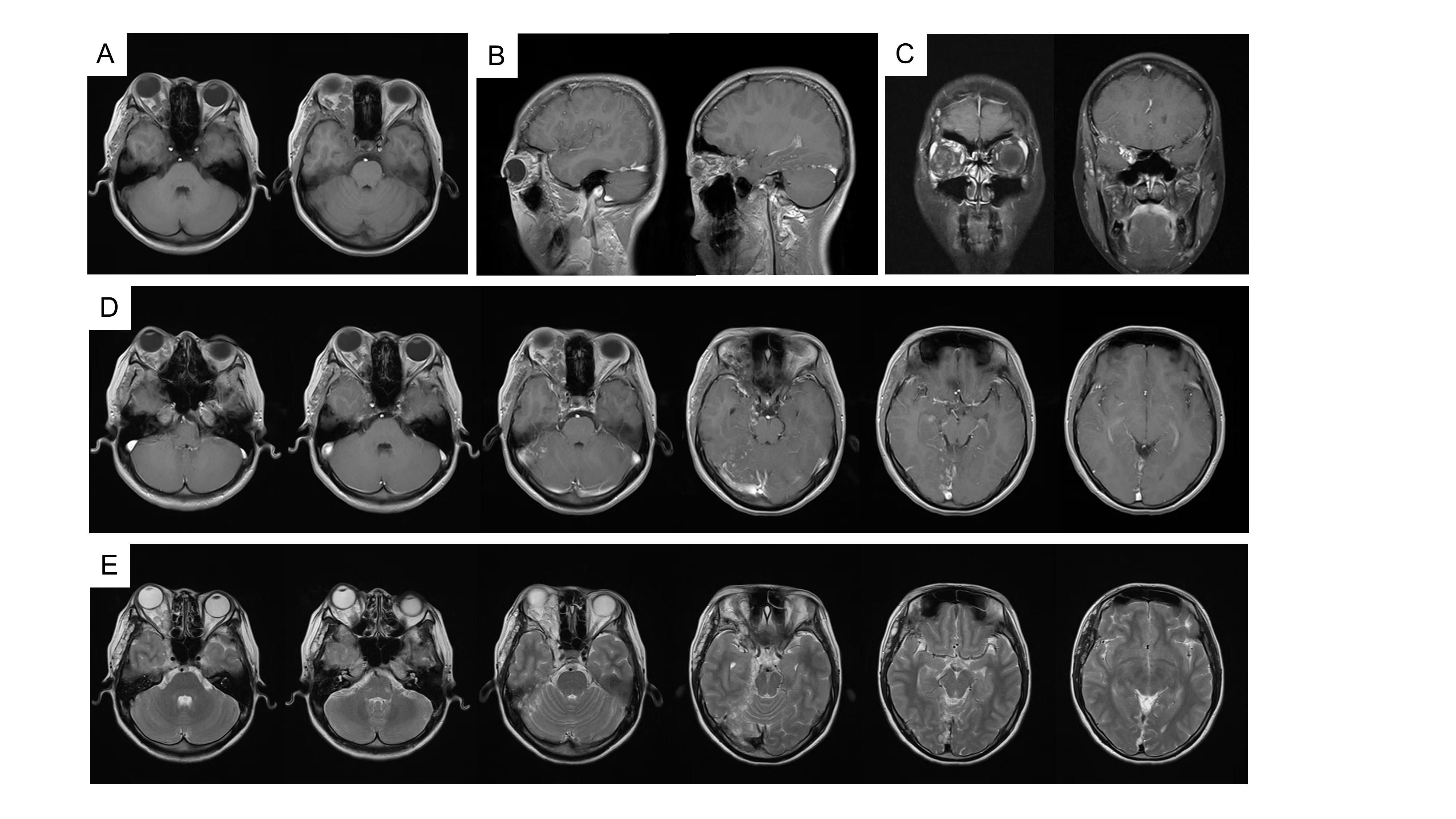


Supplementary Figure 1. MRI imaging of the first onset of the disease. (A) T1WI; (B) T1WI-C coronal view; (C) T1WI-C sagittal view; (D) T1WI-C; (E) T2WI-TSE. MRI, magnetic resonance imaging; T1WI, T1-weighted image; T2WI, T2-weighted image; T1WI-C, T1WI with contrast; T2-FLAIR, T2-fluid attenuated inversion recovery


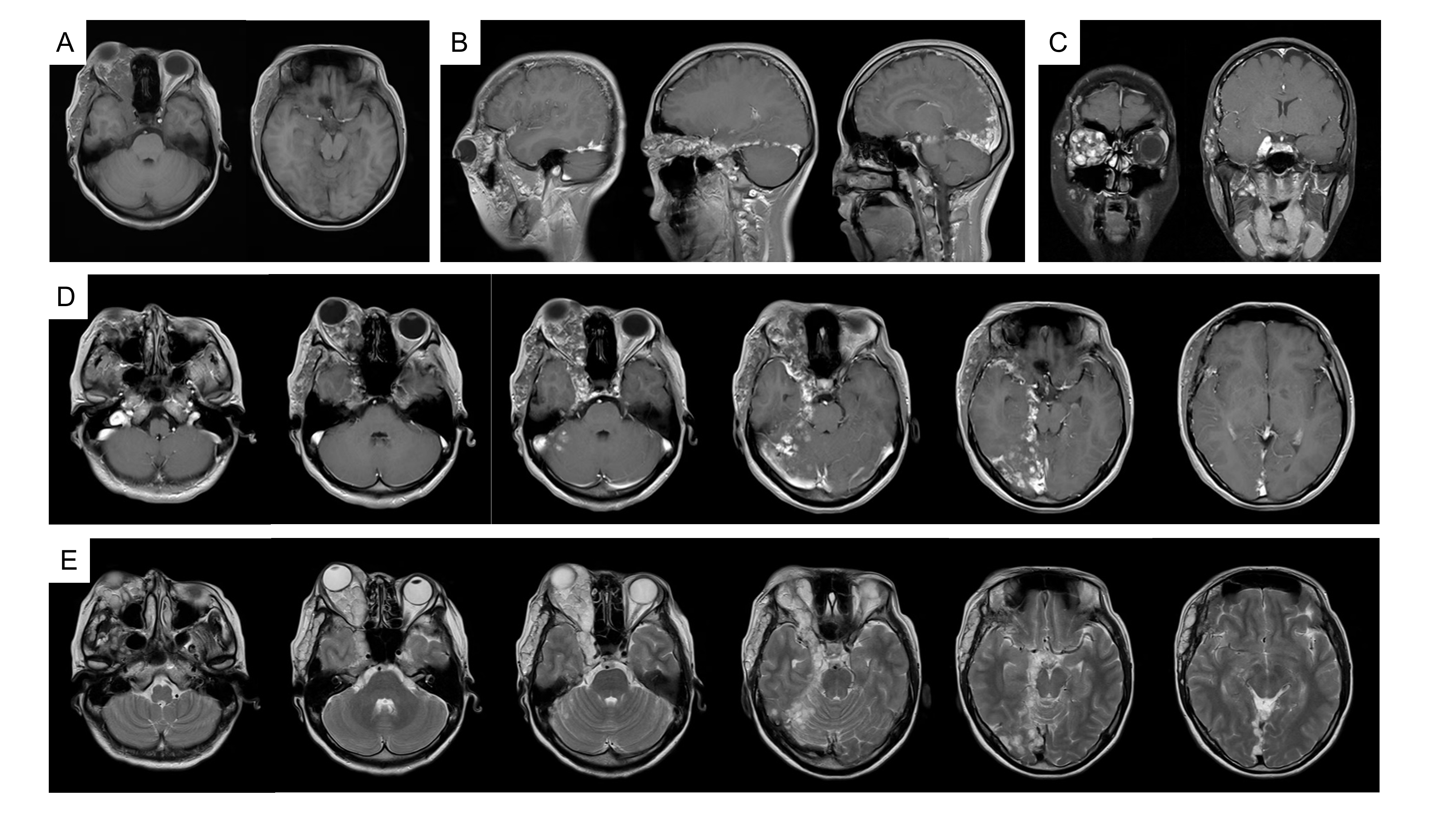


Supplementary Figure 2 MRI imaging in 2019. (A) T1WI; (B) T1WI-C coronal view; (C) T1WI-C sagittal view; (D) T1WI-C; (E) T2WI-TSE.

Supplementary Figure 3 MRI imaging of 2-month post-radiotherapy. (A) T1WI; (B) T1WI-C coronal view; (C) T1WI-C sagittal view; (D) T1WI-C; (E) T2WI-TSE.


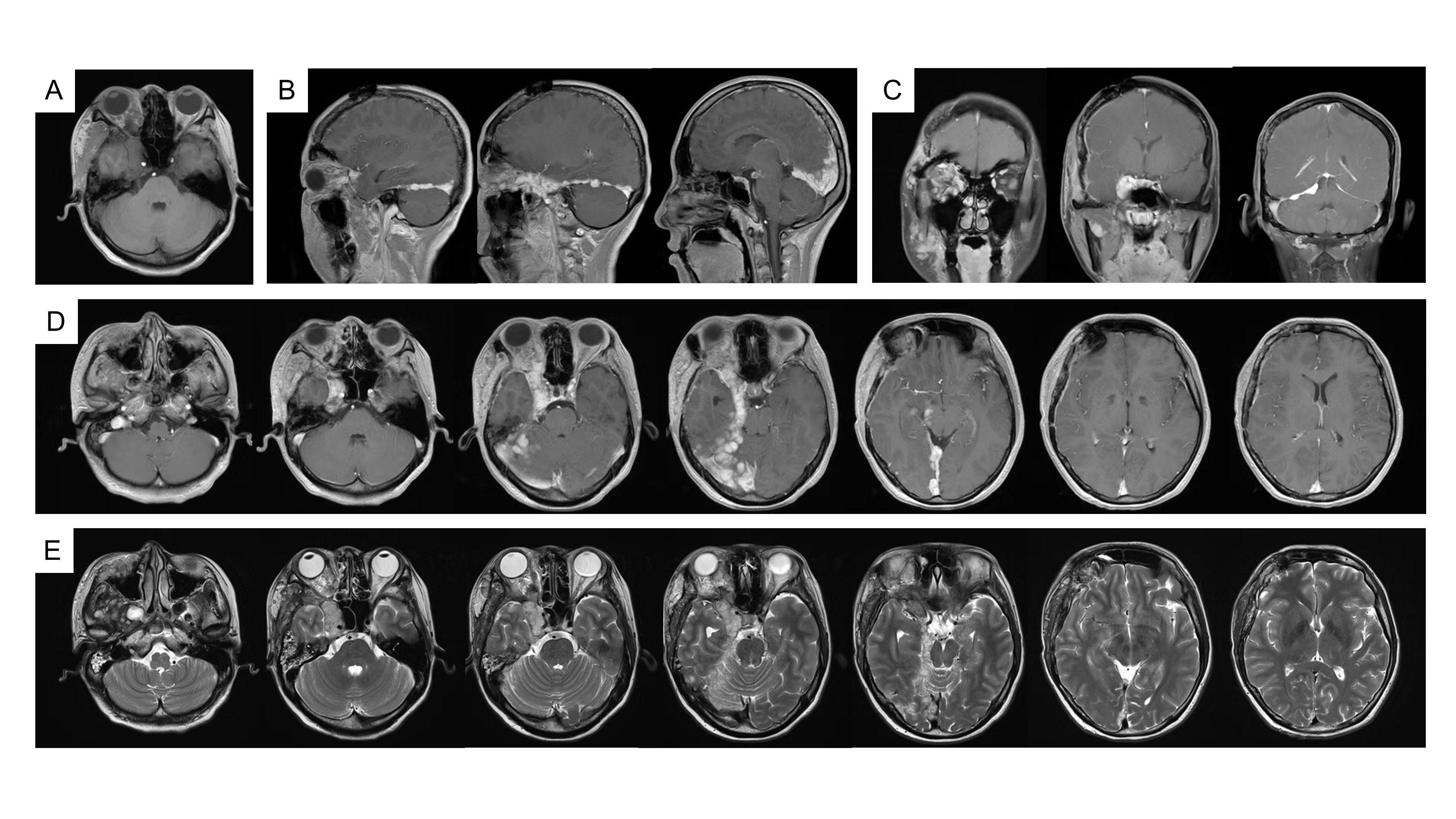

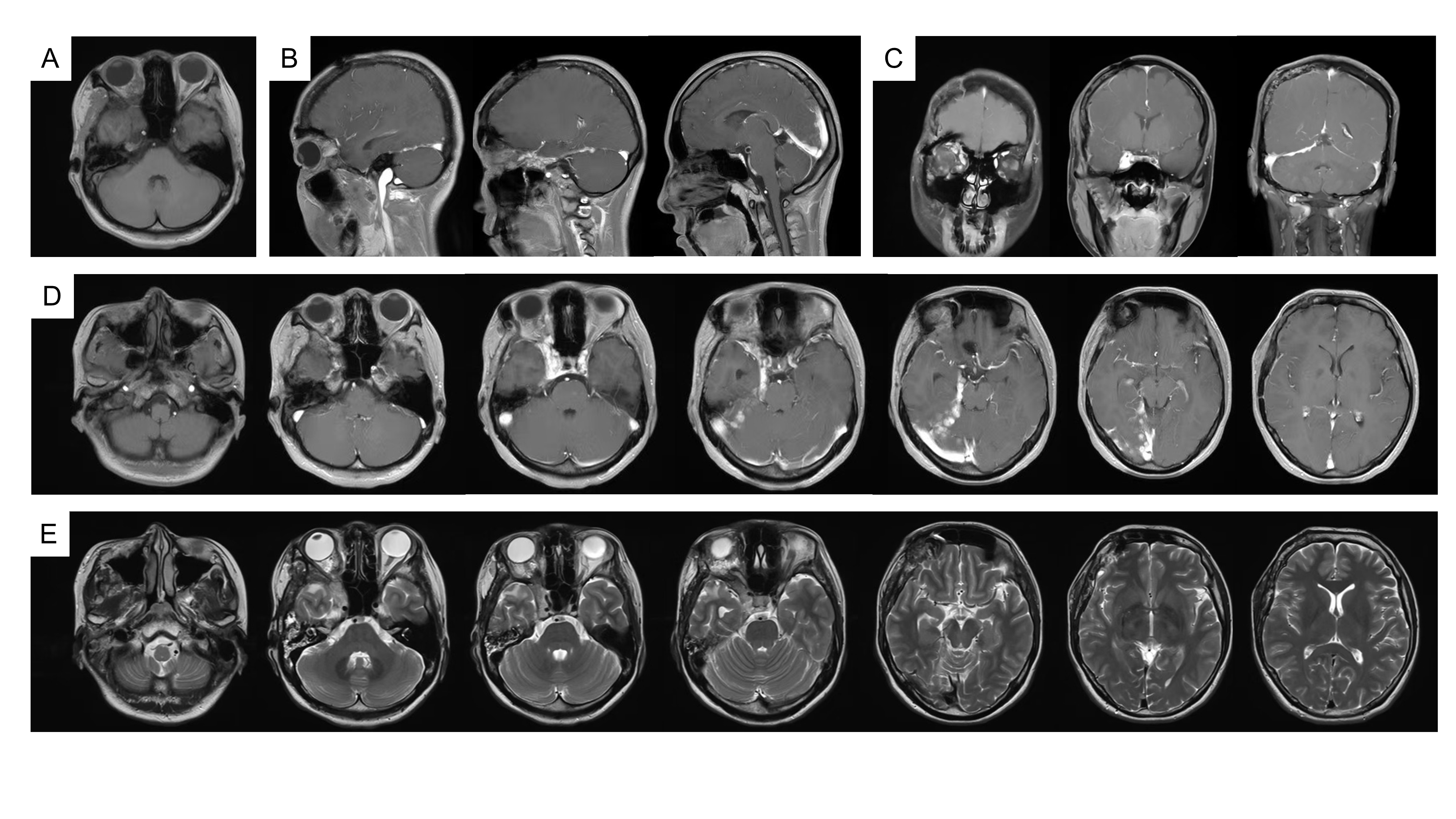


Supplementary Figure 4. MRI imaging at 6 months follow up radiology. (A) T1WI; (B) T1WI-C coronal view; (C) T1WI-C sagittal view; (D) T1WI-C; (E) T2WI-TSE.

**Supplementary materials and methods**

We utilized the Varian Eclipse 15.6 treatment planning system to perform dosimetric calculations, adhering to the dose specifications outlined in the Radiation Therapy Oncology Group (RTOG) 0933 protocol. The dosimetric data encompassed various parameters: CI, GI, HI, and OARs including the brainstem, lens, cornea, pituitary gland, retina, optic nerve and parotid.

The dosimetric parameters were calculated as follows:

1. CI_RTOG_ = PV/TV

PV, prescription volume, the volume wrapped by prescription isodose lines. TV, target volume. The higher the RTOG CI, the better the fitness.

1. CI_paddick_ = (TV_PV_×TV_PV_)/ (TV×PV)

TVPV , the volume of target area covered by prescription isodose lines. The closer the Paddick CI to 1, the better the conformal.

1. HI = D_max_/D_Dprescribed_

Dmax and Dprescribed doses were maximum and prescribed doses, respectively. The smaller the HI, the better the uniformity.

1. GI = PV_50%_/PV

PV50% is 50% isodose line wrapped volume. The lower the GI dose, the steeper the drop.
